# Comprehensive efficacy of different prostate resection volumes for patients with benign prostatic hyperplasia: a systematic review and meta-analysis

**DOI:** 10.7717/peerj.20819

**Published:** 2026-02-16

**Authors:** Puze Wang, Jinze Li, Ziqiao Tang, Liangren Liu, Dong Lv

**Affiliations:** 1 Department of Urology, People’s Hospital of Deyang City, Deyang, Sichuan Province, China; 2 Department of Oral Medicine, People’s Hospital of Deyang City, Deyang, Sichuan Province, China; 3 Department of Urology, West China Hospital of Sichuan University, Chengdu, Sichuan Province, China

**Keywords:** Multiple surgical approaches, Complications, Systematic review, Benign prostatic hyperplasia, Prostate resection volumes, Urinary-related outcomes, Perioperative outcomes, Multiple complication outcomes, Meta-analysis

## Abstract

Benign prostatic hyperplasia is one of the most common urological diseases in middle-aged and elderly men worldwide. The most effective treatment is surgery, and multiple surgical approaches, including traditional electroprostatectomy, laser vaporization, or steam ablation, have already been widely applied in clinical practice. However, few studies summarizing and reporting the comprehensive outcomes of whether different prostate resection volumes affect the efficacy of benign prostatic hyperplasia surgery exist. Thus, we conducted a systematic review and meta-analysis involving cohort studies and randomized controlled trials to compare the postoperative influence of different residual prostate volumes in patients with benign prostatic hyperplasia (BPH) to explore the best surgical treatment and minimize the recurrence rate and other complications. A total of 16 randomized controlled trials (RCTs) involving 2,164 patients who underwent prostate surgery were included in our analysis. In summary, compared with patients who had a smaller resected prostate volume, patients with more resected prostate tissue were more likely to have a lower International Prostate Symptom Score (IPSS), a lower postvoid residual urine volume (PVR), a higher quality of life (QoL) and maximal urinary flow rate (Qmax), and a decreased risk of bladder neck construction. However, patients with less resected prostate volume had advantages in terms of decreasing catheterization time, hospital stay, irrigation time, and the rates of blood transfusion and retrograde ejaculation. Moreover, the resected prostate volume was not correlated with the incidence of other complications. Given the limitations existing in our study, more primary studies are still needed in the future.

## Introduction

Benign prostatic hyperplasia (BPH) is a common disease worldwide and refers to the nonmalignant hyperplasia of prostate tissue. Studies have shown that patients with BPH account for nearly half of all men older than fifty years, and its prevalence could be as high as eighty percent in those older than seventy years of age (Ng, Leslie & Baradhi, 2024). Technically, the term BPH can be reserved only for histological diagnosis rather than for the description of clinical disease. Thus, other definitions, including bposit prostatic obstruction (BPO) or benign prostatic enlargement (BPE), are widely used in clinical work, especially for patients with lower urinary tract symptoms (LUTs) and bladder outlet obstruction (BOO) (Abrams et al., 2003; Gratzke et al., 2015). The urologist assesses the severity of BPH on the basis of the International Prostate Symptom Score (IPSS) and selects the appropriate treatment, such as medication and lifestyle behavior therapy (Barry et al., 2017). For patients who do not respond adequately to conservative treatment, surgery can be an effective option. Common surgical methods include traditional transurethral resection of the prostate (TURP), transurethral laser vaporization, plasma kinetic transurethral resection of the prostate (PK-TURP), or prostatic steam ablation. Among these procedures, TURP is considered the gold standard for prostate surgery and, to this day, continues to be ubiquitously employed. All these procedures can significantly alleviate LUTS in patients, especially during the voiding phase (Miernik & Gratzke, 2020; Martinelli et al., 2023). During the surgical procedure, the total prostate resection volume can be variable and is usually determined by the surgeon. The minimal TURP involves only localized tunneling resection, whereas the maximal TURP removes all glandular tissue down to the surgical capsule (Shin et al., 2023). Complications following urethral prostate surgery include bleeding, bladder neck contracture, urethral stricture, bladder perforation, and temporary difficulty urinating, which are possibly associated with the volume of prostate tissue removed during the operation (Ottaiano et al., 2022; Park et al., 2012). However, few studies have compared whether different prostate resection volumes can affect the incidence of postoperative complications in patients diagnosed with BPH. Therefore, we conducted this systematic review and meta-analysis to determine the impact of prostate resection volume on functional outcomes and complication rates in patients undergoing surgery for BPH.

## Materials and Methods

### Literature research

Our prospective study followed the Preferred Reporting Items for Systematic Reviews and Meta-Analysis 2020 statement (PRISMA, Table S1) and PROSPERO registries (number: CRD42024411008). Primary studies reporting the associations between prostate resection volume and postoperative complications were systematically conducted by using electronic databases, including PubMed, Embase, Web of Science and the Cochrane Library, up to October 1st, 2025. The following items were used in our search: “prostatic hyperplasia”, “surgery”, “prostate volume” and “complications”. The specific search strategy is shown in Table S2.

### Identification of eligible studies

The following studies were considered eligible: (1) the study design was a randomized controlled trial (RCT); (2) the study included patients who underwent prostatectomy; (3) the study included prostate resection volumes and baseline prostate volume; (4) at least one complication was reported; and (5) sufficient data were available to calculate odds ratios (ORs) or weighted mean differences (WMDs). Reviews, letters, editorial comments, case reports, unpublished articles, study protocols, and non-English articles were excluded. In addition, if the relationships of prostate resection volume or weight between the different groups were not significantly different, the primary studies were also excluded. For different primary studies enrolling participants from the same database, we included only the study with the largest sample size if they had the same experimental timing and inclusion criteria. In addition, references meeting our criteria from similar meta-analyses were also included in this study.

### Data extraction

Two investigators (Puze Wang and Jinze Li) performed the data extraction independently, and a third investigator (Dong Lv) made a final decision if there were any disagreements. Data for the following aspects were recorded if possible: (1) basic literature information, such as first author, publication year, study period, country of study, study design, sample size, type of surgery, and center numbers; (2) demographic data, such as age, sex, preoperative comorbidities (such as organ dysfunction and diabetes), preoperative prostate volume, and urodynamic test results; and (3) urinary outcomes, perioperative outcomes and complication outcomes, including but not limited to IPSS, quality of life (QoL), maximal urinary flow rate (Qmax), PVR, catheterization time, hospital stay, infections, TUR syndrome, bladder neck undermining, bladder perforation, bladder neck contractures (BNCs), pain, urethral strictures, retrograde ejaculation and refractory overactive bladder symptoms (OABS). If studies reported continuous variables such as the median with range or interquartile range, we used the validated mathematical method to obtain a concrete mean ± standard deviation (Luo et al., 2018; Wan et al., 2014).

### Quality assessment

Our analysis utilized the Cochrane risk of bias assessment tool (CRBAT) to evaluate the quality of the included RCTs (Higgins et al., 2011). In addition, the evidence level for every study was also assessed according to the Oxford Centre for Evidence-Based Medicine Levels of Evidence Working Group (Howick et al., 2011). Evaluations were independently performed by the same two investigators, and any discrepancies were reported to the same third investigator and finally resolved through discussion. All related information was shown in Table S3.

### Statistical analysis and publication bias

Review Manager version 5.4 (Cochrane Collaboration, Oxford, UK) was used to perform evidence synthesis. We disposed of dichotomous and continuous variables applied for OR and WMD and used 95% confidence intervals (CIs) to report all metrics; the chi-square (*χ*^2^) test and inconsistency index (I^2^) were used to assess any potential heterogeneities in our included studies. If the *χ*^2^ *P* value was < 0.05 or I^2^ was > 50%, heterogeneity was considered significant. When significant heterogeneity was detected, a random effects model was used to estimate the combined WMD or OR. In addition, one-way sensitivity analyses were performed to estimate the effects of studies on the combined results for outcomes if obvious heterogeneity was detected. Otherwise, publication bias was evaluated visually *via* funnel plots *via* Review Manager version 5.4.

## Results

### Literature search and study characteristics

**Figure 1:**
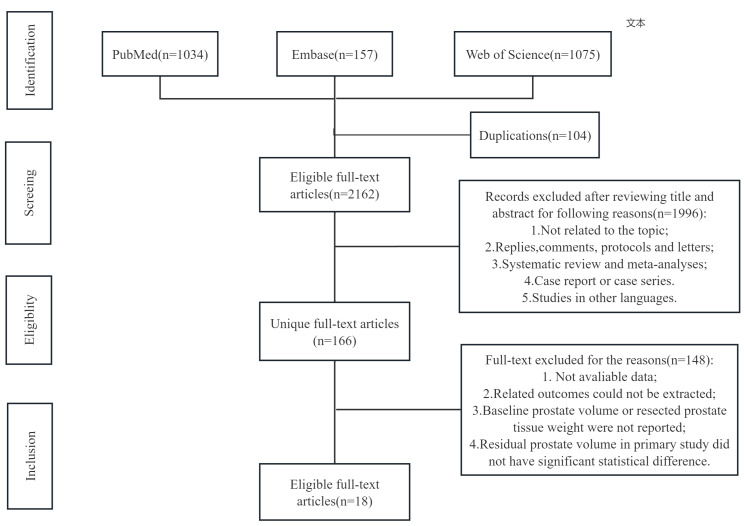
Flowchart.

The flowchart of the literature selection process is presented in Fig. 1. A total of 2,266 relevant primary studies in PubMed (*n* = 1,034), Embase (*n* = 157) and Web of Science (*n* = 1,075) were first identified through our systematic and artificial searches. A total of 166 unique studies were selected for the second screening after excluding 2,100 articles by reviewing their titles and abstracts. Finally, 16 eligible full-text randomized control trials involving 1,806 patients who underwent prostate surgery were included in this meta-analysis (Enikeev et al., 2020; Geavlete et al., 2015; Sun et al., 2019; Liu et al., 2006; Kuntz & Lehrich, 2002; Zhu et al., 2013; Xie et al., 2014; Helke et al., 2001; Zhao et al., 2010; Sun et al., 2014; Naspro et al., 2006; Ou et al., 2010; Mavuduru et al., 2009; Mattiasson et al., 2007; Jahnson et al., 1998; Dahlstrand et al., 1995). If studies involved more than two types of prostatic surgery, we conducted comparisons between every two types of surgery independently by repeating the inclusion of the same study. In addition, the mean prostate volume for each group was estimated based on baseline and postoperative prostate data, and subgroup analyses were also conducted according to the difference in average prostate volume between the two groups. In this study, the group with the larger resection volume was designated as the control group. Most of the involved studies were evaluated as medium or high-quality studies according to our assessment. The baseline characteristics of the included studies were shown in Table 1. In addition, the results of forest plot and funnel plot were also documented in Figs. S1 and S2.

### Urination-related outcomes

#### International prostate symptom score *(IPSS)*

Fourteen primary studies involving 5,739 patients reported IPSSs at different follow-up times.

##### 0%–10% difference in prostate volume.

No significant difference was detected between the two groups for postoperative IPSSs of 3 and 6 months (3 months: three studies; WMD = 0.29; 95% CI [−0.22, 0.80]; *p* = 0.27; 6 months: two studies, WMD = 0.20; 95% CI [−0.28, 0.68]; *p* = 0.42; however, for 12 months of follow-up, patients with a less resected prostate had a higher IPSS than those with more resected prostate tissue did (three studies; WMD = 0.50; 95% CI [0.07–0.93]; *p* = 0.02). In addition, the funnel plot did not indicate apparent publication bias in the primary studies.

##### 10%–20% difference in prostate volume.

Our analysis revealed that when the follow-up time was less than 1 year, no significant difference was detected for the IPSSs of these two groups (3 months: two studies; WMD = −0.43; 95% CI [−1.23–0.37]; *p* = 0.29; 6 months: three studies, WMD = 0.14; 95% CI [−0.39–0.67]; *p* = 0.61; 12 months: four studies; WMD = 0.20; 95% CI [−0.29–0.69]; *p* = 0.42). However, for an IPSS greater than 1 year, patients whose prostate tissue was less removed had a greater IPSS than did the other patients (24 months: three studies; WMD = 1.30; 95% CI [0.84–1.76]; *p* < 0.00001; *I*^2^ = 86%; 36 months: three studies, WMD = 1.25; 95% CI [0.78–1.72]; *p* < 0.00001; *I*^2^ = 92%), with significant heterogeneity. No significant publication bias was found from the funnel plot.

**Table 1:** Details of involved studies.

Study	Simple size (n)	Country	Surgery type		Resected volume of prostate (Mean ± SD)		Perioperative volume of prostate (Mean ± SD)		Difference percentage
			Experimental group	Control group	Experimental group	Control group	Experimental group	Control group	
Enikeev et al. (2020)	134	Russia	TURP	Monopolar enucleation	49.0 ± 11.1	43.6 ± 11.8	62.3 ± 13.0	59.0 ± 14.3	0–10
Geavlete et al. (2015)	320	Romania	BPEP TURis TUVis	OP	98.7 ± 41.9	110.3 ± 43.7	122.6 ± 30.7	128.7 ± 32.7	0–10
Sun et al. (2019)	115	China	Thulium laser resection	Thulium laser enucleation	4.1 ± 1.9	2.2 ± 1.2	26.4 ± 3.5	25.1 ± 4.7	0–10
Liu et al. (2006)	76	China	TUVR	TURP	32.28 ± 7.1	35.58 ± 4.3	60.58 ± 10.9	58.48 ± 8.4	0–10
Kuntz & Lehrich (2002)	120	Germary	Holmium laser enucleation	OP	83.9 ± 21.9	96.4 ± 36.4	114.6 ± 21.6	113.0 ± 19.2	10–20
Zhu et al. (2013)	80	China	TURP	Enucleation	64.2 ± 19.0	50.6 ± 20.0	113.8 ± 32.0	109.4 ± 32.4	10–20
Xie et al. (2014)	90	China	TURP	EPLA	65.3 ± 13.8	49.0 ± 12.7	93.3 ± 14.8	96.6 ± 12.1	10–20
Helke et al. (2001)	185	Germany	TUVRP	TURP	21.98 ± 13.47	30.37 ± 22.01	48.8 ± 21.21	49.9 ± 22.1	10–20
Zhao et al. (2010)	204	China	TURP	Enucleation	56.4 ± 12.8	43.8 ± 15.5	69.2 ± 13.5	67.5 ± 11.8	10–20
Sun et al. (2014)	164	China	TURP	Holmium laser enucleation	44.43 ± 19.98	32.96 ± 15.84	55.11 ± 29.03	56.22 ± 30.48	20–30
Naspro et al. (2006)	80	Italy	Holmium laser enucleation	OP	59.33 ± 34.77	87.90 ± 41.11	89.40 ± 32.82	93.39 ± 30.64	20–30
Ou et al. (2010)	69	China	TURP	Transvesical Prostatectomy	116.8 ± 33.2	69.7 ± 24.9	138.4 ± 35.3	131.0 ± 36.1	30–40
Mavuduru et al. (2009)	30	India	Holmium laser enucleation	TURP	6.53 ± 0.52	20 ± 1.66	36.53 ± 12.33	36.33 ± 11.4	30–40
Mattiasson et al. (2007)	145	Sweden	TUMT	TURP	0	NA	49 ± 16	53 ± 17	>50%^*^
Jahnson et al. (1998)	85	Sweden	TUIP	TURP	0	20.3 ± 8.5	26.7 ± 4.02	26.3 ± 4.55	>50%
Dahlstrand et al. (1995)	69	Sweden	TUMT	TURP	0	17 ± 10	NA	NA	>50%^*^

**Notes.**

TURP transurethral resection of the prostate BPEP bipolar plasma enucleation of the prostate TURis transurethral resection in saline TUVis transurethral vaporization in saline OP open prostatectomy TUVR transurethral electrovapor resection EPLA extraperitoneal laparoscopic adenomectomy TUVRP transurethral vaporesection of the prostate TUMT transurethral microwave thermotherapy TUIP Transurethral incision of the prostate NA not avaliable SD standard difference

* The difference between perioperative and postoperative prostate volume was estimated as more than 50 percent.

##### 20%–30% difference in prostate volume.

A higher IPSS was observed in the group with 20–30% less resected prostate volume than in the control group at the 3-, 6- and 12-month follow-ups (3 months: three studies; WMD = 0.54; 95% CI [0.08–1.00]; *p* = 0.02; 6 months: three studies, WMD = 0.70; 95% CI [0.32–1.08]; *p* = 0.0003; 12 months: three studies; WMD = 0.92; 95% CI [0.59–1.25]; *p* < 0.00001), with no significant heterogeneity (*I*^2^ = 0). No significant publication bias was detected in the funnel plot.

##### 30%–40% difference in prostate volume.

Two studies involving 99 patients reported that when the difference in resected prostate tissue was 30%–40%, patients with a less resected prostate had a lower IPSS than those with a more resected prostate during the 3-month follow-up (WMD = −2.84; 95% CI [−3.51 to −2.17]; *p* < 0.00001), with significant heterogeneity (*I*^2^ = 95). In addition, the funnel plot did not report significant publication bias.

##### >50% difference in prostate volume.

For the 3-, 24- and 60-month follow-ups, we detected no significant difference in the IPSS between these two groups (3 months: three studies; WMD = 0.01; 95% CI [−0.37, 0.39]; *p* = 0.96; 24 months: two studies; WMD = 0.85; 95% CI [−0.34–2.03]; *p* = 0.16; 60 months: two studies; WMD = 0.11; 95% CI [−1.15–1.38]; *p* = 0.86). However, at the 6- and 12-month follow-ups, patients with a greater proportion of prostate tissue resected had a lower IPSS than did the other groups of patients did (6 months: three studies; WMD = 0.33; 95% CI [0.01–0.64]; *p* = 0.04; 12 months: three studies; WMD = 0.38; 95% CI [0.10–0.65]; *p* = 0.007), with no significant heterogeneity. In addition, the funnel plot did not indicate apparent publication bias in the primary studies.

#### Quality of life (QOL)

A total of 10 articles with 196 involved patients reported the QOL scores of different groups.

##### 0%–10% difference in prostate volume.

Our results revealed no significant difference in postoperative QOL at any of the follow-up months (3 months: three studies; WMD = −0.05; 95% CI [−0.16–0.06]; *p* = 0.36; 6 months: two studies; WMD = −0.06; 95% CI [−0.16–0.03]; *p* = 0.16; 12 months: three studies; WMD = −0.04; 95% CI [−0.10–0.03]; *p* = 0.27). Moreover, the funnel plot did not report apparent publication bias.

##### 10%–20% difference in prostate volume.

Our results revealed that when the follow-up time was shorter than 1 year, there were no correlations between these two groups (3 months: two studies; WMD = 0.20; 95% CI [−0.11–0.51]; *p* = 0.21; 6 months: three studies, WMD = 0.08; 95% CI [−0.02–0.17]; *p* = 0.12; 12 months: three studies; WMD = 0.06; 95% CI [−0.04–0.15]; *p* = 0.23). However, when the follow-up time was longer than 1 year, patients with 10%–20% less resected prostate tissue had higher QOL scores than patients in the control group did (24 months: three studies; WMD = 1.30; 95% CI [0.84–1.76]; *p* < 0.00001; 36 months: three studies, WMD = 1.25; 95% CI [0.78–1.72]; *p* < 0.00001), with significant heterogeneity (*I*^2^ = 86%; *I*^2^ = 92%). In addition, no significant publication bias was found from the funnel plot.

##### 20%–30% difference in prostate volume.

We found that prostate surgery with 20%–30% more resected volume had advantages in increasing the QOL score in patients during the 6-month follow-up (one study, WMD = −0.13; 95% CI [−0.22, −0.04]; *p* = 0.003). Interestingly, our results revealed that patients with less resected prostate tissue had a greater quality of life than patients with 20%–30% more resected tissue in the early follow-up stage did (3 months; two studies; WMD = 0.23; 95% CI [0.12–0.35]; *p* < 0.0001). However, no significant relationship was observed between those two groups at the 12-month follow-up (three studies; WMD = −0.04; 95% CI [−0.12–0.03]; *p* = 0.28). Moreover, the funnel plot did not indicate significant publication bias in the primary studies.

##### >50% difference in prostate volume.

Our results revealed a significant correlation between the two groups: if the difference in the percentage of prostate volume removed was greater than 50%, patients with less resected prostate tissue had a worse quality of life than patients with more resected tissue within a year did (3 months: two studies; WMD = −0.25; 95% CI [−0.34, −0.17]; *p* < 0.00001; 6 months: two studies; WMD = −0.28; 95% CI [−0.34, −0.22]; *p* < 0.00001; 12 months: three studies; WMD = −0.15; 95% CI [−0.21, −0.08]; *p* < 0.00001), with significant heterogeneity. In addition, the funnel plot did not reveal apparent publication bias.

#### Maximal urinary flow rate (Qmax) (mL/s)

Twelve primary RCTs involving 5,521 patients reported the Qmax results.

##### 0%–10% difference in prostate volume.

There was no significant difference in the Qmax between the two groups within a year of follow-up (3 months: three studies; WMD = −0.49; 95% CI [−1.22–0.24]; *p* = 0.19; 6 months: two studies; WMD = 0.21; 95% CI [−0.81–1.23]; *p* = 0.68; 12 months: three studies; WMD = −0.22; 95% CI [−0.90–0.46]; *p* = 0.52). Moreover, the funnel plot revealed slight publication bias in the results at the 3- and 6-month follow-ups.

##### 10%–20% difference in prostate volume.

Our results indicated that patients with 10–20% more resected prostate had advantages in improving postoperative Qmax (6 months: three studies; WMD = −1.13; 95% CI [−2.19, −0.07]; *p* = 0.04; 12 months: four studies, WMD = −1.48; 95% CI [−2.54, −0.41]; *p* = 0.007; 24 months: three studies; WMD = −4.21; 95% CI [−5.43, −2.99]; *p* < 0.00001; 36 months: three studies; WMD = −4.05; 95% CI [−5.48, −2.62]; *p* < 0.00001) except at the 3-month follow-up (3 months: two studies; WMD = 0.72; 95% CI [−0.52–1.97]; *p* = 0.25). In addition, no significant heterogeneity was detected in the results of more than 1 year of follow-up (*I*^2^ = 0%). In addition, no significant publication bias was reported according to the funnel plot.

##### 20%–30% difference in prostate volume.

Our results revealed no significant difference in the postoperative Qmax between the two groups at 3 and 6 months (3 months: two studies; WMD = −0.63; 95% CI [−1.38–0.11]; *p* = 0.1; 6 months: one study; WMD = −0.66; 95% CI [−1.43–0.12]; *p* = 0.1). Moreover, patients with more resected tissue were observed to have a greater Qmax during the 12-month follow-up (12 months: three studies; WMD = −0.86; 95% CI [−1.54, −0.18]; *p* < 0.00001), with no significant heterogeneity (*I*^2^ = 0%). In addition, slight publication bias could be detected from the funnel plot.

##### >50% difference in prostate volume.

We found that there was a significant relationship between the two groups in that patients with a greater resected prostate volume had a greater postoperative Qmax than patients in the control group did (3 months: three studies; WMD = −2.72; 95% CI [−3.30, −2.13]; *p* < 0.00001; 6 months: three studies; WMD = −2.70; 95% CI [−3.29, −2.12]; *p* < 0.00001; 12 months: three studies; WMD = −2.90; 95% CI [−3.48, −2.31]; *p* < 0.00001; 24 months: two studies; WMD = −4.48; 95% CI [−6.55, −2.40]; *p* < 0.0001). However, most of the results presented obvious heterogeneity, with the exception of the results at the 24-month follow-up. Moreover, the funnel plot revealed slight publication bias in these results.

#### Postvoid residual urine volume (PVR) (mL)

Eleven primary studies involving 4,908 patients reported PVR results.

##### 0%–10% difference in prostate volume.

Our study revealed that there was a significant relationship between the two groups in that patients with greater resected prostate volume had greater advantages in improving PVR than patients in the control group at 3-, 6- and 12-month follow-ups did (3 months: three studies; WMD = −4.65; 95% CI [−8.12–−1.17]; *p* = 0.009; 6 months: two studies; WMD = −7.47; 95% CI [−10.37–−4.57]; *p* < 0.00001; 12 months: three studies; WMD = −3.01; 95% CI [−5.09–−0.93]; *p* = 0.004). In addition, no significant publication bias was revealed from the funnel plot.

##### 10%–20% difference in prostate volume.

There was a significant correlation between the two groups in that patients with a smaller resected prostate volume had greater PVR than patients in the control group at all follow-up months, with apparent heterogeneity (3 months: two studies; WMD = 1.09; 95% CI [0.24–1.94]; *p* = 0.01; 6 months: three studies; WMD = 0.57; 95% CI [0.23–0.92]; *p* = 0.001; 12 months: three studies; WMD = 0.68; 95% CI [0.28–1.08]; *p* = 0.0009; 24 months: three studies; WMD = 0.74; 95% CI [0.39–1.09]; *p* < 0.0001; 36 months: two studies; WMD = 3.40; 95% CI [2.83–3.97]; *p* < 0.00001). In addition, the funnel plot revealed no significant publication bias in these results.

##### 20%–30% difference in prostate volume.

Our study revealed that patients with 20%–30% less resected prostate volume had a greater PVR than patients in the control group did during the 12-month follow-up (12 months: two studies; WMD = 6.36; 95% CI [4.33–8.38]; *p* < 0.00001). However, no significant difference was detected between the 3- and 6-month follow-up groups (3 months: one study; WMD = −1.05; 95% CI [−4.29–2.20]; *p* = 0.53; 6 months: one study; WMD = −1.93; 95% CI [−4.19–0.33]; *p* = 0.09). Moreover, slight publication bias was revealed from the funnel plot.

##### >50% difference in prostate volume.

No significant difference was detected between the two groups in terms of the postoperative PVR at 3 and 12 months of follow-up (3 months: one study; WMD = −2.23; 95% CI [−4.68–0.22]; *p* = 0.07; 12 months: two studies; WMD = 1.49; 95% CI [−0.26–3.23]; *p* = 0.09). At the 6-month follow-up, patients with less prostate reserve also had a lower PVR than patients in the other groups did (6 months: two studies; WMD = 6.35; 95% CI [4.23–8.47]; *p* < 0.00001). A funnel plot also revealed slight publication bias in the results.

### Perioperative outcomes

#### Catheterization time (days)

In total, 13 RCTs involving 2,237 patients reported the postoperative catheterization time. Subgroup analysis revealed that patients with 30%–40% greater resected prostate volume had longer catheterization times than patients in the other groups did at all follow-up months, with no heterogeneity (WMD = −2.14; 95% CI [−2.64–−1.64]; *p* < 0.00001, *I*^2^ = 0). However, subgroup analysis also revealed that there was no significant difference between groups with other differential prostate volumes. In addition, the funnel plot revealed slight publication bias.

#### Hospital stay (days)

In total, nine articles involving 1,943 patients reported the length of hospital stay. The results revealed that patients who underwent prostate resection had a shorter hospital stay, with significant heterogeneity (WMD = −0.61; 95% CI [−0.69, −0.53]; *p* < 0.00001, *I*^2^ = 99%). A funnel plot also revealed low-grade publication bias.

#### Irrigation length (days)

Two primary studies involving 990 patients reported that patients with fewer resected prostates had a shorter irrigation length, with significant heterogeneity (WMD = −0.47; 95% CI [−0.54, −0.41]; *p* < 0.00001, *I*^2^ = 99%). Publication bias was not apparent according to the results of the funnel plot.

### Complication outcomes

#### Urethral stricture

Ten RCTs, including 1,082 participants, reported that patients with different resected prostates had similar incidences of postoperative urethral stricture, with no heterogeneity (WMD = 1.49; 95% CI [0.75–2.95]; *p* = 0.25, *I*^2^ = 0%). However, a visual assessment of the funnel plot indicated significant publication bias among these patients.

#### Urinary retention

Pooled analysis of five studies involving 538 patients revealed no significant difference in the incidence of UR between patients with different resected prostate tissues (WMD = 2.25; 95% CI [0.99–5.15]; *p* = 0.84, *I*^2^ = 0%). Moreover, the funnel plot indicated negative publication bias in the primary studies.

#### Urinary incontinence

In total, nine studies involving 917 patients reported no significant differences between the two groups of participants with different resected prostate volumes (WMD = 0.84; 95% CI [0.50–1.40]; *p* = 0.68, *I*^2^ = 0%). In addition, the funnel plot suggested significant publication bias.

#### Urinary tract infection

Data on the incidence of urinary tract infection were synthesized from 6 studies involving 731 participants. Both total and subgroup analyses suggested that the incidence of urinary tract infection was similar between the two groups (WMD = 1.07; 95% CI [0.55–2.10], *p* = 0.83; *I*^2^ = 0%). Moreover, obvious publication bias could be observed in the funnel plot.

#### Prolonged bladder irrigation

Two studies involving 254 patients reported the outcome of prolonged bladder irrigation. Our results revealed that no significant difference was detected between the two groups whose reserved prostate was less than 20% (WMD = 1.06; 95% CI [0.39–2.93], *p* = 0.91, *I*^2^ = 0%). In addition, no significant publication bias was observed in the funnel plot.

#### Clot retention

Three RCTs involving 428 participants reported that the incidence of postoperative clot retention was similar between the two groups (WMD = 1.54; 95% CI [0.68–3.48], *p* = 0.30; *I*^2^ = 0%). In addition, the funnel plot suggested significant publication bias.

#### Bladder neck sclerosis

A total of three primary studies involving 344 patients reported the incidence of bladder neck sclerosis, and no significant differences were detected between the two groups of participants with less than 30% different resected prostate volumes (WMD = 0.67; 95% CI [0.15–3.13], *p* = 0.62, *I*^2^ = 0%). Moreover, the funnel plot indicated negative publication bias in the primary studies.

#### Blood transfusion

In total, data on blood transfusion after prostate surgery were extracted from 10 studies with 1,868 patients. No significant difference or heterogeneity was detected in the forest plot when the difference in prostate volume was less than 50% (WMD = 0.65; 95% CI [0.39–1.09], *p* = 0.1, *I*^2^ = 48%). However, our results suggest that if the difference in resected prostate tissue was greater than 50%, patients with more resected prostate tissue had a higher rate of blood transfusion than those with less resected prostate tissue did (WMD = 0.18; 95% CI [0.04–0.79], *p* = 0.02, *I*^2^ = 0%). However, most of the outcomes were observed to have apparent publication bias according to the funnel plot.

#### Recatheterization

No obvious difference was detected between the two groups of patients whose difference in removed prostate volume was less than 30% according to the recatheterization data from two studies involving 196 participants, with no heterogeneity (WMD = 0.88; 95% CI [0.25–3.12], *p* = 0.84, *I*^2^ = 0%). In addition, publication bias was not apparent in the funnel plot.

#### Recurrence

No significant difference or heterogeneity was detected in the forest plot of the rate of recurrence between the two groups (WMD = 1.28; 95% CI [0.26–6.24], *p* = 0.76; *I*^2^ = 0%). Slight publication bias was observed in the funnel plot.

#### BNCs

A total of three primary studies involving 409 patients revealed that when the difference in the removed prostate volume was less than 30%, patients lost with more prostate tissue had a decreased risk of BNCs with no heterogeneity (WMD = 2.88; 95% CI [1.14–7.26], *p* = 0.03, I2=0%). In addition, the funnel plot suggested slight publication bias.

#### Retrograde ejaculation

Data on postoperative retrograde ejaculation were extracted from three studies with 370 patients. When the difference in removed prostate volume was less than 30%, patients with more reserved prostate fluid had a higher incidence of etrograde ejaculation, with significant heterogeneity (WMD = 2.41; 95% CI [1.52–3.85], *p* = 0.0002, *I*^2^ = 86%). A funnel plot revealed obvious publication bias.

#### Transurethral resection syndrome

Two studies involving 280 participants reported the outcome of postoperative transurethral resection syndrome. No significant differences were detected between the two groups of participants whose resected prostate volume was less than 30% different (WMD = 0.86; 95% CI [0.18–4.15], *p* = 0.85; *I*^2^ = 63%). In addition, the funnel plot did not suggest publication bias.

### Sensitivity analysis

Sensitivity analysis was conducted to resolve the existence of heterogeneity in outcomes by excluding studies in the series through Review Manager 5.4. The results revealed that significant heterogeneity of retrograde ejaculation was eliminated by excluding Xie’s study, and the result changed after heterogeneity was resolved in that there was no obvious correlation between those two groups (WMD = 1.19; 95% CI [0.68–2.06], *p* = 0.54, *I*^2^ = 0%). Moreover, heterogeneity in other outcomes could not be eliminated by excluding any involved primary studies.

## Discussion

At present, although minimally invasive therapies are on the rise, TURP is still the most widely used surgery for treating BPH, and it is considered the gold standard for the treatment of LUTS (Sciacqua et al., 2023; Akpayak et al., 2017). Most TURP aims at debulking the prostate to produce an adequate channel for urine to flow by reducing the volume of the middle or lateral lobes of the prostate. However, owing to differences in the cognition and habits of surgeons, the proportion of actual resected prostate tissue produced by different surgeries or the same surgery could vary in clinical practice. Although various devices can be used for TURP, including electrocautery loops, lasers, and steam, it is believed that there are no uniform limits for the size and shape of these prostate resections (Leslie, Chargui & Stormont, 2024). Several minimally invasive procedures, including water vapor thermal infusion or microwave thermotherapy, induce localized destruction of prostate tissue and can ultimately reduce the prostate volume by approximately 30% (Mynderse et al., 2015; Franco et al., 2022).

Our study focused on the following three important indicators of prostatectomy: urinary, perioperative and complication outcomes. The urination-related outcomes are divided into several subgroups, including IPSS, QoL, Qmax and PVR. The IPSS is an effective and widely used tool for evaluating patients’ self-reported symptoms, as recommended by the American Urological Association. According to our research, in the subgroup analysis of different proportions of resected prostates, patients with more reserved tissue had a higher IPSS, which indicates a lower level of LUT alleviation. A study involving 667 participants reported a positive correlation between the IPSS and urethral resistance (Huang Foen Chung & Van Mastrigt, 2005). The larger the volume of prostate resection is, the greater the reduction in urethral resistance, leading to a decrease in the patient’s IPSS score. Furthermore, we found that among patients with a prostate resection volume difference greater than 50%, those with less preserved prostate tissue generally had a higher QoL; for patients with a difference of less than 50%, the impact of prostate resection on QoL remains a subject of debate. Specifically, although our study could not establish a significant correlation between resected prostate volume and QOL, the original studies to varying degrees indicate the definite advantage of prostate surgery over conservative treatment in improving patients’ quality of life (Geavlete et al., 2015; Sun et al., 2019; Liu et al., 2006; Zhu et al., 2013; Xie et al., 2014). A previous study demonstrated that other voiding parameters, including the Qmax and PVR, are also common tools used to evaluate postoperative voiding functions and have statistically significant relationships with IPSS (Kohler & Kausik, 2023). We found that when the difference in prostate volume resected exceeded 10%, patients with a larger resected volume had a greater Qmax than did those with a smaller volume, and the results were statistically significant. Conversely, when the difference in resected prostate volume ranged between 10% and 40%, the residual urine volume decreased as the resected volume increased. However, owing to the limited number of original studies, no significant difference in PVR was observed between patient groups with a resected prostate volume difference greater than 50% (Geavlete et al., 2015).

The catheterization time for patients who undergo transurethral resection or ablation is generally within 7 days, with the optimal time typically considered to be the third postoperative day. For patients who experience difficulty urinating after catheter removal and undergo recatheterization, the appropriate timing for subsequent removal of the catheter is four days later (Skolarus et al., 2019). Our results indicated that there was no significant correlation between catheterization time and the volume of prostate resection among the majority of patients. However, for the 30%–40% group, patients with more resected prostate tissue had longer catheterization times. The possible reason for this was that the original studies involving this group compared transvesical prostatectomy, which is associated with greater trauma and blood loss (Ou et al., 2010). Similarly, patients with a greater proportion of resected prostate tissue also had a longer irrigation time and hospital stay.

Our review also consolidated and analyzed the multiple types of complications reported in the included studies as much as possible. The most common complication of BPH surgery is bleeding, which mostly occurs during the operation procedure and the early postoperative period. Severe bleeding is more likely to occur in traumatic prostate surgeries, such as traditional open surgery and transurethral prostate resection. According to previous studies, the transfusion rate of monopolar TURP has historically reached approximately 0.4%–20% (Teo, Lee & Ho, 2017). However, with advancements in surgical techniques and equipment, the rate of blood transfusion has significantly decreased in contemporary practice, which is similar to our findings. For patients on anticoagulant medication, the risk of bleeding and blood transfusion are increased, and appropriate multidisciplinary consultations are necessary during the perioperative period (Heiman, Large & Krambeck, 2018; Romero-Otero et al., 2019). In addition, the other positive complication was retrograde ejaculation. Our research revealed that when the removed prostate volume was less than 30%, patients with more reserved prostate fluid had a higher incidence of etrograde ejaculation, with significant heterogeneity. It is generally believed that retrograde ejaculation is caused by damage to the bladder neck and sympathetic nerves during prostate surgery (Meng et al., 2007; Shoshany et al., 2017). Research has demonstrated that preserving the verumontanum and one centimeter of proximal tissue and mucosa during prostatectomy and preserving the bladder neck can significantly reduce the incidence of retrograde ejaculation (Alloussi et al., 2014; Abolazm et al., 2020; Yang et al., 2023). In general, our study revealed that there was no significant relationship between the overall incidence of complications and the volume of prostate resection. Complications of the procedure might be related to age, comorbidities, preoperative duration and multiple factors. Furthermore, the perioperative prostate volume and prolonged catheterization could also result in an increased rate of complications (Geremew, Gelaw & Beyene, 2022). Additionally, the duration of the operation is another important factor influencing postoperative complications. Studies have shown that a long duration promotes the rate of capsular perforation and transfusion and results in prolonged catheterization time (Tascıet al., 2011; Rassweiler et al., 2006; Mandal et al., 2013).

In addition, for a comprehensive evaluation of BPH surgical outcomes, retreatment rates for BPH procedures should not be overlooked. In general, it is reported that proportion of 1-year retreatment and 1-year complications by procedure (including TURP, photoselective vaporization of the prostate (PVP) and water vapor thermal therapy (WVTT)) is similar. However, traditional TURP has the lowest rate of 5-year treatment among cohorts of diverse surgical pethods. Otherwise, the same literature has revealed that patients accepted TURP and PVP had a higher risk of undergoing the same surgical retreatment within postoperative 30 days (Kaplan et al., 2024). Another research based on real-world data from medicare Australia reported a similar outcome for the high rate of revision surgery after TURP and PVP, but no significant difference was observed between these two groups (Jain et al., 2023).

Another aspect that warrants attention is the preservation of patients’ sexual function. Traditional prostate surgery often adversely affects sexual function, particularly ejaculatory performance of patients. Previous study has reported that the vast majority of men undergoing prostatectomy experience permanent retrograde ejaculation (Koren & Koren, 2020). Therefore, minimally invasive surgical therapies (MISTs) of prostate are continually being explored to minimize the incidence of retrograde ejaculation. The Leonardi Ejaculation Sparing Technique (LEST) is a prostate adenoma debulking technique that can be performed using either simple vaporization or enucleation for larger prostates, which can achieve postoperative antegrade ejaculation by preserving the course and openings of the ejaculatory ducts within the prostate and minimizing injury to the genital sphincter (Leonardi et al., 2025). Other MISTs, including Rezūm and i-Tind (Temporary implantable nitinol device), which aims to reshape the prostatic urethra and alleviate LUTS while preserving sexual function with favorable outcomes, have garnered significant attention in recent years (Busetto et al., 2025; Dimitri et al., 2025). Otherwise, several South Korean scholars compared the transurethral and transvesical robot-assisted simple prostatectomy, detected that the newly introduced urethral-sparing prostatectomy had clearly advantages on rapid symptom improvements and preserved antegrade ejaculation (Shin et al., 2025). However, while minimally invasive techniques continue to evolve, their widespread adoption is constrained by multifactorial barriers including socioeconomic disparities and surgical learning curves. Consequently, traditional prostatectomy remains a cornerstone approach in global practice, underscoring the imperative for rigorously designed prospective trials to evaluate integrated efficacy endpoints.

Our study is the first to explore the relationship between resected prostate volume and comprehensive postoperative efficacy, especially with respect to urinary and complication outcomes. In addition, subgroup analyses were also performed on the basis of the follow-up time and various resected prostate volumes, with the aim of exploring the long-term effects of prostate surgery. However, we must acknowledge that there are several limitations to our study. First, a part of the proportion of the resected volume was estimated roughly, which may have influenced the complication outcomes. Second, owing to the limited number of primary studies, several outcomes, including the incidence of bladder tamponade, capsular perforation or impotence, could not be analyzed. Furthermore, all RCTs explore the efficacy of two or more different surgery types. There are few clinical trials comparing the postoperative outcomes between the same surgery option and different resected prostate volumes, which may introduce bias into our results. Moreover, some outcomes in our analysis had significant noneliminated heterogeneity and publication bias, leading to inaccurate conclusions that can misguide clinical practice of BPH therapies. Thus, although our analysis revealed several meaningful outcomes, more primary studies, especially those involving the same type of surgery for BPH, are still needed in the future.

## Conclusions

In summary, our study revealed that, compared with patients who had a smaller resected prostate volume, patients with more resected prostate tissue were more likely to have a lower IPSS, a lower PVR, a higher QoL and Qmax, and a decreased risk of bladder neck construction. However, patients with less resected prostate volume had advantages in terms of decreasing catheterization time, hospital stay, irrigation time, and the rates of blood transfusion and retrograde ejaculation. Moreover, the resected prostate volume was not correlated with the incidence of other complications. Given the limitations existing in our study, more primary studies are still needed in the future.

##  Supplemental Information

10.7717/peerj.20819/supp-1 Supplemental Information 1 PRISMA (Preferred Reporting Items for Systematic Reviews and Meta-Analysis 2020 statement)

10.7717/peerj.20819/supp-2 Supplemental Information 2 The specific search strategy

10.7717/peerj.20819/supp-3 Supplemental Information 3 Quality assessment

10.7717/peerj.20819/supp-4 Supplemental Information 4 Result of forest plot

10.7717/peerj.20819/supp-5 Supplemental Information 5 Result of funnel plot

## List of abbreviations

BPH: benign prostatic hyperplasia
IPSS: International Prostate Symptom Score
QoL: quality of life
Qmax: maximal urinary flow rate
PVR: postvoid residual urine volume
RCTs: randomized controlled trials
LUTS: lower urinary tract symptoms
BPE: benign prostatic enlargement
BPO: bposit prostatic obstruction
BOO: bladder outlet obstruction
TURP: transurethral resection of the prostate
PK-TURP: plasma kinetic transurethral resection of the prostate
PRISMA: Preferred Reporting Items for Systematic Reviews and Meta-Analysis 2020 statement
ORs: odds ratios
WMDs: weighted mean differences
BNCs: bladder neck contractures
OABS: overactive bladder symptoms
CRBAT: Cochrane risk of bias assessment tool
CIs: confidence intervals
I^2^: inconsistency index
PVP: photoselective vaporization of the prostate
WVTT: water vapor thermal therapy
LEST: Leonardi Ejaculation Sparing Technique
MISTs: minimally invasive surgical therapies

## Additional Information and Declarations

### Data Availability

The following information was supplied regarding data availability: This is a Systemic Review/Meta-Analysis.

## References

[ref-1] Abolazm AE, El-Hefnawy AS, Laymon M, Shehab-El-Din AB, Elshal AM (2020). Ejaculatory hood sparing *versus* standard laser photoselective vaporization of the prostate: sexual and urodynamic assessment through a double blinded, randomized trial. Journal D Urologie.

[ref-2] Abrams P, Cardozo L, Fall M, Griffiths D, Rosier P, Ulmsten U, Van Kerrebroeck P, Victor A, Wein A, Standardization Sub-Committee of the International Continence Society (2003). The standardization of terminology in lower urinary tract function: report from the standardization subcommittee of the International Continence Society. Urology.

[ref-3] Akpayak IC, Shuaibu SI, Onowa VE, Nabasu LE, Galam ZZ (2017). Monopolar transurethral resection of the prostate for benign prostatic hyperplasia: what are the outcomes and complications in our patients?. Nigerian Journal of Medicine.

[ref-4] Alloussi SH, Lang C, Eichel R, Alloussi S (2014). Ejaculation-preserving transurethral resection of prostate and bladder neck: short- and long-term results of a new innovative resection technique. Journal of Endourology.

[ref-5] Barry MJ, Fowler FJ, O’leary MP, Bruskewitz RC, Holtgrewe HL, Mebust WK, Cockett AT, Measurement Committee of the American Urological Association (2017). The American Urological Association symptom index for benign prostatic hyperplasia. Journal of Urology.

[ref-6] Busetto GM, Lombardo R, De Nunzio C, Santoro G, Tocci E, Schiavone N, Tubaro A, Carrieri G, Kaplan SA, Herrmann TRW (2025). Ejaculation sparing of classic and minimally invasive surgical treatments of LUTS/BPH. Prostate Cancer and Prostatic Diseases.

[ref-7] Dahlstrand C, Waldén M, Geirsson G, Pettersson S (1995). Transurethral microwave thermotherapy *versus* transurethral resection for symptomatic benign prostatic obstruction: a prospective randomized study with a 2-year follow-up. British Journal of Urology.

[ref-8] Dimitri M, Calarco A, Filippi B, Viscuso P, Asero V, Mantica G, Ambrosini F, Spena G, Bucca B, Schiavina R, Piazza P, Iacono G, Tufano A, Leonardi R (2025). I-Tind for the treatment of lower urinary tract symptoms secondary to benign prostatic hyperplasia: mid-term outcomes from a multicenter cohort. Urologia.

[ref-9] Enikeev D, Rapoport L, Gazimiev M, Allenov S, Inoyatov J, Taratkin M, Laukhtina E, Sung JM, Okhunov Z, Glybochko P (2020). Monopolar enucleation *versus* transurethral resection of the prostate for small- and medium-sized (<80 cc) benign prostate hyperplasia: a prospective analysis. World Journal of Urology.

[ref-10] Franco JVA, Garegnani L, Escobar Liquitay CM, Borofsky M, Dahm P (2022). Transurethral microwave thermotherapy for benign prostatic hyperplasia: an updated cochrane review. World Journal of Men’s Health.

[ref-11] Geavlete B, Bulai C, Ene C, Checherita I, Geavlete P (2015). Bipolar vaporization, resection, and enucleation *versus* open prostatectomy: optimal treatment alternatives in large prostate cases?. Journal of Endourology.

[ref-12] Geremew LM, Gelaw SA, Beyene AD (2022). Assessing the complications of monopolar transurethral resection of the prostate (M-TURP) using Clavien–Dindo complications grading system. Ethiopian Journal of Health Sciences.

[ref-13] Gratzke C, Bachmann A, Descazeaud A, Drake MJ, Madersbacher S, Mamoulakis C, Oelke M, Tikkinen KAO, Gravas S (2015). EAU guidelines on the assessment of nonneurogenic male lower urinary tract symptoms including benign prostatic obstruction. European Urology.

[ref-14] Heiman J, Large T, Krambeck A (2018). Best practice in the management of benign prostatic hyperplasia in the patients requiring anticoagulation. Therapeutic Advances in Urology.

[ref-15] Helke C, Manseck A, Hakenberg OW, Wirth MP (2001). Is transurethral vaporesection of the prostate better than standard transurethral resection?. European Urology.

[ref-16] Higgins JP, Altman DG, Gøtzsche PC, Jüni P, Moher D, Oxman AD, Savovic J, Schulz KF, Weeks L, Sterne JA, Cochrane Bias Methods Group, Cochrane Statistical Methods Group (2011). The Cochrane Collaboration’s tool for assessing risk of bias in randomised trials. BMJ.

[ref-17] Howick J, Chalmers I, Glasziou P, Greenhalgh T, Heneghan C, Liberati A, Moschetti I, Phillips B, Thornton H (2011). Explanation of the 2011 Oxford Centre for Evidence-Based Medicine (OCEBM) levels of evidence (Background Document).

[ref-18] Huang Foen Chung JW, Van Mastrigt R (2005). Correlation of noninvasive urodynamics with international prostate symptom score (IPSS) and prostate volume. Neurourology and Urodynamics.

[ref-19] Jahnson S, Dalén M, Gustavsson G, Pedersen J (1998). Transurethral incision *versus* resection of the prostate for small to medium benign prostatic hyperplasia. British Journal of Urology.

[ref-20] Jain A, Nassour AJ, Khannani H, Wines MP, Chalasani V, Katelaris P, Bergersen P, Symons JL, Baskaranathan S, Woo H (2023). Australian surgical revision rate for benign prostatic obstruction. BJU International.

[ref-21] Kaplan S, Kaufman Jr RP, Mueller T, Elterman D, Chughtai B, Rukstalis D, Woo H, Roehrborn C (2024). Retreatment rates and postprocedural complications are higher than expected after BPH surgeries: a US healthcare claims and utilization study. Prostate Cancer and Prostatic Diseases.

[ref-22] Kohler TS, Kausik SJ (2023). Comparison of IPSS score and voiding parameters in men presenting with LUTS. The Canadian Journal of Urology.

[ref-23] Koren G, Koren D (2020). Retrograde ejaculation—a commonly unspoken aspect of prostatectomy for benign prostatic hypertrophy. American Journal of Men’s Health.

[ref-24] Kuntz RM, Lehrich K (2002). Transurethral holmium laser enucleation *versus* transvesical open enucleation for prostate adenoma greater than 100 gm.:: a randomized prospective trial of 120 patients. Journal D Urologie.

[ref-25] Leonardi R, Mantica G, Ambrosini F, Tufano A, Iacona G, Calarco A (2025). Diode laser enucleation prostate for benign prostatic hyperplasia: outcomes of the Leonardi ejaculation sparing technique. Archivio Italiano di Urologia e Andrologia.

[ref-26] Leslie SW, Chargui S, Stormont G (2024). Transurethral resection of the prostate.

[ref-27] Liu CK, Lee WK, Ko MC, Chiang HS, Wan KS (2006). Transurethral electrovapor resection *versus* standard transurethral resection treatment for a large prostate: a 2-year follow-up study conducted in Taiwan. Urologia Internationalis.

[ref-28] Luo D, Wan X, Liu J, Tong T (2018). Optimally estimating the sample mean from the sample size, median, mid-range and/or mid-quartile range. Statistical Methods in Medical Research.

[ref-29] Mandal S, Sankhwar SN, Kathpalia R, Singh MK, Kumar M, Goel A, Singh V, Sinha RJ, Singh BP, Dalela D (2013). Grading complications after transurethral resection of prostate using modified Clavien classification system and predicting complications using the Charlson comorbidity index. International Urology and Nephrology.

[ref-30] Martinelli E, Cindolo L, Grossi FS, Kuczyk MA, Siena G, Oelke M (2023). Transurethral water vapor ablation of the prostate with the Rezūm system: urodynamic findings. Neurourology and Urodynamics.

[ref-31] Mattiasson A, Wagrell L, Schelin S, Nordling J, Richthoff J, Magnusson B, Schain M, Larson T, Boyle E, Duelund-Jacobsen J, Kroyer K, Ageheim H (2007). Five-year follow-up of feedback microwave thermotherapy *versus* TURP for clinical BPH: a prospective randomized multicenter study. Urology.

[ref-32] Mavuduru RM, Mandal AK, Singh SK, Acharya N, Agarwal M, Garg S, Kumar S (2009). Comparison of HoLEP and TURP in terms of efficacy in the early postoperative period and perioperative morbidity. Urologia Internationalis.

[ref-33] Meng F, Gao B, Fu Q, Chen J, Liu Y, Shi B, Xu Z (2007). Change of sexual function in patients before and after Ho:YAG laser enucleation of the prostate. Journal of Andrology.

[ref-34] Miernik A, Gratzke C (2020). Current treatment for benign prostatic hyperplasia. Deutsches Ärzteblatt International.

[ref-35] Mynderse LA, Hanson D, Robb RA, Pacik D, Vit V, Varga G, Wagrell L, Tornblom M, Cedano ER, Woodrum DA, Dixon CM, Larson TR (2015). Rezūm system water vapor treatment for lower urinary tract symptoms/benign prostatic hyperplasia: validation of convective thermal energy transfer and characterization with magnetic resonance imaging and 3-dimensional renderings. Urology.

[ref-36] Naspro R, Suardi N, Salonia A, Scattoni V, Guazzoni G, Colombo R (2006). Holmium laser enucleation of the prostate *versus* open prostatectomy for prostates >70 g: 24-month follow-up. European Urology.

[ref-37] Ng M, Leslie SW, Baradhi KM (2024). Benign prostatic hyperplasia. StatPearls [Internet].

[ref-38] Ottaiano N, Shelton T, Sanekommu G, Benson CR (2022). Surgical complications in the management of benign prostatic hyperplasia treatment. Current Urology Reports.

[ref-39] Ou R, You M, Tang P, Chen H, Deng X, Xie K (2010). A randomized trial of transvesical prostatectomy *versus* transurethral resection of the prostate for prostate greater than 80 mL. Urology.

[ref-40] Park HK, Paick SH, Lho YS, Jun KK, Kim HG (2012). Effect of the ratio of resected tissue in comparison with the prostate transitional zone volume on voiding function improvement after transurethral resection of prostate. Urology.

[ref-41] Rassweiler J, Teber D, Kuntz R, Hofmann R (2006). Complications of transurethral resection of the prostate (TURP)—incidence, management, and prevention. European Urology.

[ref-42] Romero-Otero J, García-González L, García-Gómez B, Justo-Quintas J, García-Rojo E, González-Padilla DA, Sopeña Sutil R, Duarte-Ojeda JM, Rodríguez-Antolín A (2019). Factors influencing intraoperative blood loss in patients undergoing holmium laser enucleation of the prostate (HoLEP) for benign prostatic hyperplasia: a large multicenter analysis. Urology.

[ref-43] Sciacqua LV, Vanzulli A, Di Meo R, Pellegrino G, Lavorato R, Vitale G, Carrafiello G (2023). Minimally invasive treatment in benign prostatic hyperplasia (BPH). Technology in Cancer Research & Treatment.

[ref-44] Shin SH, Lee KS, Koo KC, Cho KS, Hong CH, Chung BH, Ryoo HS, Ryu JH, Kim YB, Yang SO, Lee JK, Jung TY, Yoo JW (2023). Effects of resection volume on postoperative micturition symptoms and retreatment after transurethral resection of the prostate. World Journal of Urology.

[ref-45] Shin YS, Pak SW, Hwang W, Jo SB, Kim JW, Oh MM, Park HS, Moon DG, Ahn ST (2025). Urethral sparing *versus* trans-vesical robot-assisted simple prostatectomy: a comparative analysis of perioperative, postoperative outcomes, and ejaculation preservation. World Journal of Men’s Health.

[ref-46] Shoshany O, Abhyankar N, Elyaguov J, Niederberger C (2017). Efficacy of treatment with pseudoephedrine in men with retrograde ejaculation. Andrology.

[ref-47] Skolarus TA, Dauw CA, Fowler KE, Mann JD, Bernstein SJ, Meddings J (2019). Catheter management after benign transurethral prostate surgery: RAND/UCLA appropriateness criteria. American Journal of Managed Care.

[ref-48] Sun N, Fu Y, Tian T, Gao J, Wang Y, Wang S, An W (2014). Holmium laser enucleation of the prostate *versus* transurethral resection of the prostate: a randomized clinical trial. International Urology and Nephrology.

[ref-49] Sun Q, Guo W, Cui D, Wang X, Ruan Y, Zhao F, Xia S, Han B, Jing Y (2019). Thulium laser enucleation *versus* thulium laser resection of the prostate for prevention of bladder neck contracture in a small prostate: a prospective randomized trial. World Journal of Urology.

[ref-50] Tascı AI, Ilbey YO, Tugcu V, Cicekler O, Cevik C, Zoroglu F (2011). Transurethral resection of the prostate with monopolar resectoscope: single-surgeon experience and long-term results of after 3,589 procedures. Urology.

[ref-51] Teo JS, Lee YM, Ho HSS (2017). An update on transurethral surgery for benign prostatic obstruction. Asian Journal of Urology.

[ref-52] Wan X, Wang W, Liu J, Tong T (2014). Estimating the sample mean and standard deviation from the sample size, median, range and/or interquartile range. BMC Medical Research Methodology.

[ref-53] Xie JB, Tan YA, Wang FL, Xuan Q, Sun YW, Xiao J, Zhu YP, Zhou LY (2014). Extraperitoneal laparoscopic adenomectomy (Madigan) *versus* bipolar transurethral resection of the prostate for benign prostatic hyperplasia greater than 80 ml: complications and functional outcomes after 3-year follow-up. Journal of Endourology.

[ref-54] Yang GR, Lü KK, Wu YY, Song T (2023). Research progress on mechanism and preventive measures of retrograde ejaculation after benign prostatic hyperplasia surgery. Zhonghua Nan Ke Xue.

[ref-55] Zhao Z, Zeng G, Zhong W, Mai Z, Zeng S, Tao X (2010). A prospective, randomized trial comparing plasmakinetic enucleation to standard transurethral resection of the prostate for symptomatic benign prostatic hyperplasia: three-year follow-up results. European Urology.

[ref-56] Zhu L, Chen S, Yang S, Wu M, Ge R, Wu W, Liao L, Tan J (2013). Electrosurgical enucleation *versus* bipolar transurethral resection for prostates larger than 70 ml: a prospective, randomized trial with 5-year followup. Journal D Urologie.

