# Supplementary material for: Comprehensive efficacy of different prostate resection volumes for patients with benign prostatic hyperplasia: a systematic review and meta-analysis
Source: PeerJ. 2026 Feb 16;14:e20819. doi: 10.7717/peerj.20819 (PMC12919313; doi:10.7717/peerj.20819)
Supplement: Supplemental Information 2 [file peerj-14-20819-s002.docx]

Pubmed

("prostatic hyperplasia"[MeSH Terms] AND ("volum"[All Fields] OR "volume"[All Fields] OR "volumes"[All Fields] OR "voluming"[All Fields])) AND (clinicaltrial[Filter])

WOS

(benign prostatic hyperplasia) AND (volume) (Topic) and Preprint Citation Index (Exclude – Database) and Clinical Trial (Document Types)

Embase

#1=(benign prostatic hyperplasia and volume).mp. [mp=title, abstract, heading word, drug trade name, original title, device manufacturer, drug manufacturer, device trade name, keyword heading word, floating subheading word, candidate term word]

limit 1 to (full text and human and english language and (embase or medline) and clinical trial and english and article)
