# Supplementary material for: Comprehensive efficacy of different prostate resection volumes for patients with benign prostatic hyperplasia: a systematic review and meta-analysis
Source: PeerJ. 2026 Feb 16;14:e20819. doi: 10.7717/peerj.20819 (PMC12919313; doi:10.7717/peerj.20819)
Supplement: Supplemental Information 3 [file peerj-14-20819-s003.docx]

|  | Random sequence generation | Allocation concealment | Blinding of participants and personnel | Blinding of outcome assessment | Incomplete outcome data | Selective reporting | Other bias | Evidence of Oxford Centre |
| --- | --- | --- | --- | --- | --- | --- | --- | --- |
| Enikeev, 2019 | + | + | + | ? | + | - | ? | 1b |
| Geavlete, 2015 | + | ? | + | + | + | + | + | 1b |
| Sun. 2019 | + | ? | - | - | + | + | + | 2b |
| Liu, 2006 | + | ? | - | - | + | ? | ? | 2b |
| Rainer M, 2002 | ? | + | ? | ? | + | + | ? | 2b |
| Zhu, 2013 | + | + | - | - | + | + | - | 2b |
| Xie, 2014 | ? | + | ? | ? | + | - | - | 2b |
| Helke, 2001 | + | ? | ? | - | + | + | - | 2b |
| Zhao, 2010 | + | + | - | - | + | + | ? | 2b |
| Sun, 2014 | + | + | - | - | + | + | + | 2b |
| Richard, 2006 | ? | + | ? | - | + | + | - | 2b |
| Ou, 2010 | + | ? | - | - | + | + | ? | 2b |
| Mavuduru, 2009 | + | + | ? | ? | + | + | - | 2b |
| Anders. 2007 | + | + | - | - | + | ? | - | 2b |
| Jahnson S, 1998 | ? | + | - | - | + | + | - | 2b |
| Dahlstrand C, 1995 | + | ? | - | - | + | + | - | 2b |
| +: Low risk of bias  -: High risk of bias  ?: unclear risk of bias. | | | | | | | |  |

**Supplementary Table 3**: Assessment of the quality of the included RCTs.
