# Supplementary figures and images for: Comprehensive efficacy of different prostate resection volumes for patients with benign prostatic hyperplasia: a systematic review and meta-analysis
Source: PeerJ. 2026 Feb 16;14:e20819. doi: 10.7717/peerj.20819 (PMC12919313; doi:10.7717/peerj.20819)

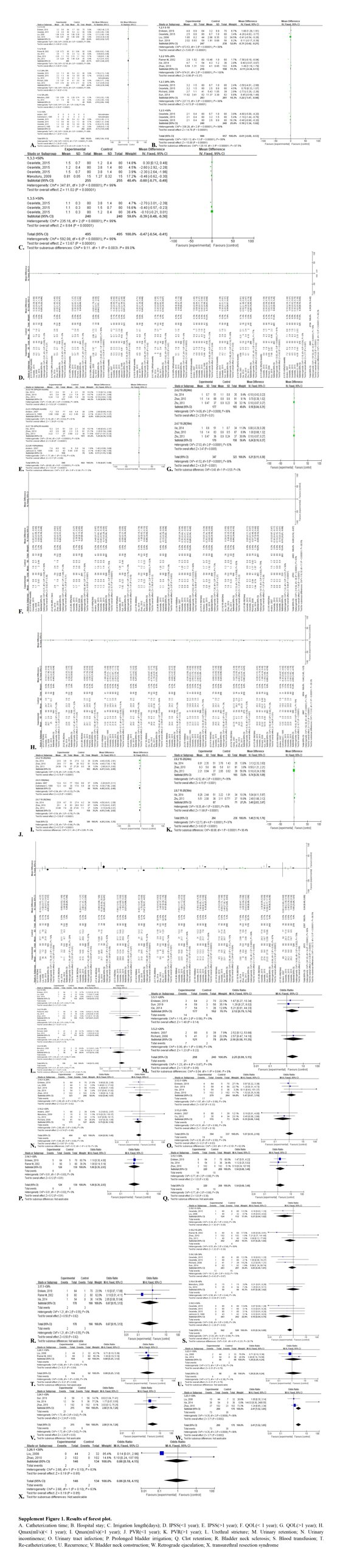

Supplement: Supplemental Information 4 [file peerj-14-20819-s004.png]

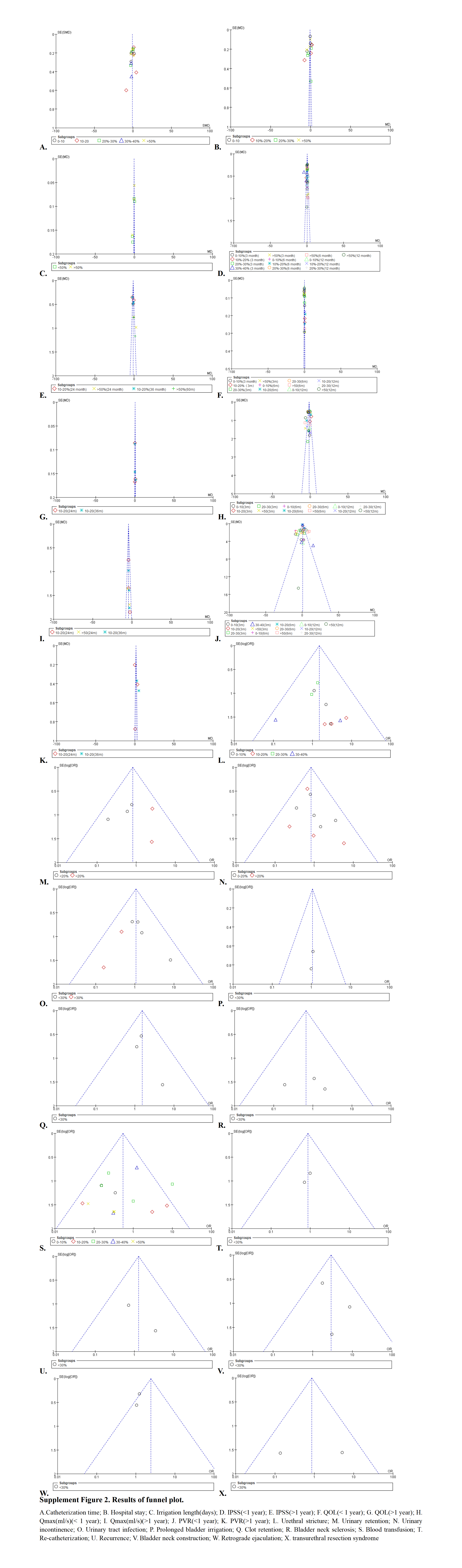

Supplement: Supplemental Information 5 [file peerj-14-20819-s005.png]
